# Metabolic syndrome and periodontal disease

**DOI:** 10.4103/0972-124X.60234

**Published:** 2009

**Authors:** Vipin Bharti, Pankaj Khurana

**Affiliations:** *Department of Periodontology, Government Dental College and Hospital, Patiala, Punjab, India*; 1 *Department of Periodontology, Government Dental College and Hospital, Patiala, Punjab, India*

**Keywords:** Cardiovascular disease, insulin resistance, metabolic syndrome, periodontal disease, type 2 diabetes obesity

## Abstract

It is important for a dentist to be well informed and updated on the latest research on the association of oral and systemic health. Of late, the metabolic syndrome has gained importance in dental literature, and metabolic syndrome and periodontal disease have been linked. Metabolic syndrome (MeS) is a group of three or more (up to five) interrelated metabolic abnormalities, which increases the risk of cardiovascular morbidity and mortality. Also, both MeS and periodontal disease may be linked through a common pathophysiological pathway. Some studies have been conducted to show such an association and additional studies are required to establish this association. A dental surgeon can play a major role in evaluating patients with MeS and thus prevent the development of overt cardiovascular disease.

## INTRODUCTION

Hardly a day goes by in the world of dentistry without some mention of oral systemic relationship. It is important for the dentist to be well informed about the latest research in oral systemic relationship and implement appropriate plans for its prevention and treatment. Of late, metabolic syndrome (MeS) and periodontal disease have been linked. According to the Adult Treatment Panel III and recent consensus workshops, MeS is defined as the concurrence of not only hypertension and atherogenic lipid profiles (hypertriglyceridemia and low density lipoproteins), but also obesity and insulin resistance. A proinflammatory and procoagulant state, with elevation of C-reactive protein and fibrinogen coexist in this syndrome. Also, periodontal diseases have been associated with systemic alteration such as, low grade inflammation, dyslipidemia, a procoagulant state, glucose intolerance, and endothelial dysfunction. Therefore, both MeS and periodontal disease may be linked through a common pathophysiological pathway.[1]

## METABOLIC SYNDROME IS WORTH CARING ABOUT

MeS is a condition that can pave the way to type 2 diabetes mellitus and cardiovascular disease. A diagnosis of MeS is associated with doubling the risk for future cardiovascular diseases and type 2 diabetes mellitus.[1] Thus, MeS is one of the major public issues of our time. It is known by different names — Syndrome X, Deadly Quartet, insulin resistance syndrome, and plurimetabolic syndrome.[2]

## DEFINITION AND DIAGNOSIS OF METABOLIC SYNDROME

Important definitions[3] have been proposed by the World Health Organization (WHO) and the European Group for the Study of Insulin Resistance (EGIR) in 1999, National Cholesterol Education Program Adult Treatment Panel III (NCEP ATP) in 2001, and the latest by the International Diabetic Federation (IDF) in 2005 [Tables 1 and 2].

**Table 1:** Diagnosis of metabolic syndrome as proposed by the international diabetes federation[4]

Person must have
Central obesity (defined by waist circumference) - Specific values for different ethnic groups given in Table 2 below
Plus any two or more of the following four factors-
Raised triglyceride levels — ≥ 150 mg/dl (≥ 1.7 mmol/L) or specific treatment for this lipid abnormality. Reduced high density lipoproteins levels — ≤ 40 mg/dl (≤ 1.03 mmol/L) in males and ≤ 50 mg/dl (≤ 1.29 mmol/L) in females, or specific treatment for this lipid abnormality. Raised blood pressure (BP) — systolic blood pressure ≥ 130 mm Hg or diastolic blood pressure ≥ 85 mmHg or treatment of previously diagnosed hypertension. Raised fasting blood glucose levels — ≥ 100 mg/dl (≥ 5.6 mmol/L) or treatment of previously diagnosed type 2 diabetes.

**Table 2:** Ethnic specific values for waist circumference

Country/ethnic group	Waist circumference	

	Females	Males
Europids*	≥ 94 cm (38 inches)	≥ 80 cm (32 inches)
South Asians	≥ 90 cm (36 inches)	≥ 80 cm (32 inches)
Chinese	≥ 90 cm (36 inches)	≥ 80 cm (32 inches)
Japanese	≥ 85 cm (34 inches)	≥ 90 cm (36 inches)
Ethnic South and Central Americans	Use South Asian recommendations until more specific data are available.	
Sub-Saharan Africans	Use European data until more specific data are available.	
Eastern Mediterranean. and Middle East (Arab) populations	Use European data until more specific data are available	

* (In USA, for Europids, Adult treatment Panel III values (102 cm.males, 88 cm females) are likely to continue for clinical purposes)

## EPIDEMIOLOGY

The study results based on the Third National Health and Nutrition Examination Survey (NHANES III) indicate that approximately one-fourth of the US adults, 20 years or older, meet the diagnostic criteria for MeS. Looking at various studies around the world, which include population samples, aged 20-25 and upwards, the prevalence varies from 8 (India) to 24% (United States) in men and from 7 (France) to 46% (India) in women.[4]

MeS is common among Arab populations in Mediterranean countries and among Arab Americans. In Jordan, a cross-sectional study showed a prevalence of 36.3% of MeS.[5]

## AETIOPATHOGENESIS

Many factors affect the pathogenesis of MeS. Age, gender, ethnicity, diet, physical activity, various unknown genetic, and endocrine factors, all play a role. However, the pathogenesis of MeS is poorly understood, although, insulin resistance and central obesity are fundamental.[6]

A simple model of MeS would include low level of exercise and high dietary intake, leading to body fat accumulation (Obesity). Obesity is associated with increased insulin resistance [Table 3]. The abundant free fatty acids (FFAs) from expanded adipose tissue decrease insulin sensitivity in muscles. As a result, there is diminished glucose disposal, hyperglycemia, and pancreatic beta cell stimulation to produce extra insulin (hyperinsulinemia). The residual FFAs, which are not absorbed in the muscle cells, are diverted to the liver via the portal vein. In the liver, these FFAs alter lipoprotein production, leading to dyslipidemia. The putative link between hyperinsulinemia and hypertension may be through sympathetic nervous system stimulation, causing vasoconstriction and increased renal Na reabsorption.[7]

**Table 3:** Proposed pathogenesis of the metabolic syndrome[7]

Imbalance between energy intake and energy expenditure		
↓		
Increased waist circumference		
↓		
Adipocytes unable to take up glucose and store free fatty acids (FFAs)		
↓		
FFAs are released into systemic circulation		
↓		
Muscle cells take up few of the FFAs.	→ Excess FFAs diverted to liver	
↓	↓	
Muscle cells develop insulin resistance.	FFAs alter lipoprotein production in liver	
↓	↓	
Hyperglycemia	Triglycerides level	↑
	HDL level	↓
↓		
Pancreas produce additional insulin (Hyperinsulinemia)		
↓		
Sympathetic nervous system stimulated		
↓.→ Hypertension		

Also, adipose tissue, secretes numerous immunomodulatory factors called ADIPOKINES that promote the risk factors of MeS [Table 4].[8,9]

**Table 4:** Role of adipokines in promoting risk factors of metabolic syndrome

Leptin suppress appetite and increase energy expenditure. Most obese persons have elevated leptin levels that do not suppress appetite. Leptin may elevate blood pressure and contribute to atherosclerosis and cardiovascular disease. Adiponectin has anti-atherogenic properties and improves insulin sensitivity. Its levels are decreased in obese subjects. Tumor necrosis factor-alpha and interleukin-6 may increase insulin resistance by preventing the auto-phosphorylation of the insulin receptor and suppressing second messenger signaling, through the inhibition of the enzyme tyrosinase kinase. Plaminogen activator inhibitor-1 prevents the dissolution of clots by inhibiting extracellular matrix degradation and fibrinolysis. It is thought to contribute to the development of type 2 diabetes and coronary thrombi. Angiotensinogen has vasoconstrictive effects on the blood vessels, and contributes to hypertension. Vascular endothelial growth factor plays a role in atherogenesis and hypertension.

## PERIODONTAL DISEASE AND METABOLIC SYNDROME

Meta-analyses have shown the association of periodontal diseases and cardiovascular diseases. There is growing evidence that suggests an association among periodontal diseases and the risk factors for cardiovascular disease, including obesity,[10,11] dyslipidemia,[12,13] diabetes mellitus,[14] and hypertension.[15] Few studies are carried out if the accumulation of the above risk factors in the form of MeS increases the risk of periodontal disease. As described earlier, both the metabolic syndrome and periodontal disease are associated with systemic inflammation, insulin resistance, and endothelial dysfunction; these two diseases are linked through a common pathophysiological pathway. This could explain the almost 20% increase risk of cardiovascular disease reported in patients with periodontitis.[1]

Nishmura *et al*,[16] proposed that periodontal disease should be considered a part of MeS. Shimazaki *et al*,[17] conducted a periodontal examination on 584 women with MeS and found that women with more components of MeS had a significantly higher odds ratio for greater probing depth and clinical attachment loss.

D'Aiuto *et al*,[1] examined 13,994 men and women in a cross-sectional survey. The prevalence of the MeS was 18, 34, and 37% among individuals with mild, moderate, and severe periodontitis, respectively.

Khader *et al*,[5] conducted a study in 78 patients with MeS and 78 patients without MeS. Results showed that the percentage of sites with clinical attachment loss and probing depth ≥3 mm were significantly greater among patients with MeS. Percentage of sites with clinical attachment loss ≥3 mm increased with an increase in components of MeS.

Additional large-scale longitudinal, epidemiological, and intervention studies are needed to establish the association of the MeS and periodontal disease.

## MANAGEMENT OF METABOLIC SYNDROME

The International Diabetes federation (IDF) recommended, “Management of the MeS should be aggressive and uncompromising in its aim to reduce the risk of cardiovascular disease and type 2 diabetes. Patients should undergo a full cardiovascular risk assessment”.[4] Similarly, in 2005, the AHA/NHLBI (American Heart Association/National Heart, Lung and Blood Institute) scientific statement recommended, “to reduce lifetime risk for atherosclerotic cardiovascular disease, all individuals found to have the metabolic syndrome deserve long-term management and follow-up in the clinical setting.”[18]

The prime emphasis in individuals with MeS is to mitigate the underlying risk factors (obesity, physical inactivity, and atherogenic diet) through lifestyle changes. In people for whom lifestyle changes is not enough, drug therapy may be required. It is necessary to treat individual components of MeS in order to reduce the individual risk associated with every single component.

Dental surgeons can also play a great role in evaluating such patients. Dental surgeons treating patients with medical histories and physical signs indicating possible metabolic disorder such as increased waist circumference as well as hypertension should ask a patient if he or she has ever been evaluated for MeS or its component risk factors, because early comprehensive intervention may prevent the development of overt cardiovascular disease.[7] If the patient is not under a physician's care, the dental surgeon should refer him or her for proper evaluation and treatment.

## CONCLUSION

MeS is a significant predictor of cardiovascular disease and type 2 diabetes. Early intervention by the physician as well as dental surgeon can prevent the development of overt cardiac problems. The underlying biological mechanisms showing a relation between MeS and periodontal disease remains to be established and more studies are required to prove this association.
